# Study on the correlation between the severity of patellofemoral arthritis and the morphology of the distal femur

**DOI:** 10.1186/s12891-023-06198-z

**Published:** 2023-02-02

**Authors:** Chongyi Fan, Yingzhen Niu, Maozheng Wei, Lingce Kong, Fei Wang

**Affiliations:** Department of Joint Surgery, Hebei Medical University Affiliated Third Hospital, Shijiazhuang, 050051 Hebei China

**Keywords:** Patellofemoral osteoarthritis, Distal femoral torsion, Patellar tilt, CT

## Abstract

**Purpose:**

Distal femoral torsion is a key factor for poor alignment of patellofemoral joint. This study aims to evaluate the correlation between distal femoral torsion and the severity of patellofemoral arthritis, and to analyze the correlation between distal femoral torsion and the morphology of femoral condyle.

**Methods:**

A retrospective analysis was performed on 125 patients awaiting surgical treatment for knee osteoarthritis from January 2021 to March 2022(79 females, 46 males, average age: 65.78 years, SD 6.61). All patients underwent knee joint radiography, lower-limb digital radiography, and knee joint CT scans. The ratio of length of each distal femoral condyle, TT-TG, patellar tilt, DFL-PCL, DFL-TEA, TEA-PCL and TEA-ACL were measured. The Pearson correlation coefficient was used to evaluate the correlation between distal femoral torsion and ratio of distal femoral condyle, TT-TG and patellar tilt. Logistic regression was used to evaluate the correlation between each parameter and the severity of PFOA.

**Results:**

With the increased severity of PFOA, TT-TG, patellar tilt, DFL-PCL, DFL-TEA and PCA all tended to increase. Patellar tilt was correlated with DFL-PCL (*r* = 0.243) and TEA-PCL(*r* = 0.201), but TT-TG had no evident correlation with distal femoral torsion. Compared with Grade I patients of PFOA, DFL-PCL, DFL-TEA, and TEA-PCL were risk factors for increased severity of patellofemoral arthritis in Grade III patients of PFOA, but there was no significant statistic difference in Grade II patients of PFOA.

**Conclusions:**

Distal femoral torsion correlates with the severity of patellofemoral arthritis. Variation of the femoral transepicondylar axis caused by the change of ratio of the femoral condyle is particularly important in the distal femoral torsion. In patients with severe PFOA, abnormal variation of the femoral condyle axis should be not ignored.

**Supplementary Information:**

The online version contains supplementary material available at 10.1186/s12891-023-06198-z.

## Background

There are many risk factors for patellofemoral osteoarthritis (PFOA), including patellar dislocation, abnormal lower-limb force line, abnormal lower-limb rotary force line, trochlea dysplasia, muscle strength and ligament injury [1]. Trochlear dysplasia has been widely reported as an important risk factor for patellofemoral arthritis [2]. Meanwhile, patients with trochlear dysplasia are most likely to develop the deformation within the distal posterior femoral condyle. Studies by Yang et al. demonstrated a more prominent anterolateral condyle and a shorter posterolateral condyle in patients with patellar dislocation and trochlear dysplasia [3]. Julien Roger et al. have also recently confirmed that the shortening of the posterolateral condyle is associated with trochlea dysplasia and patellar dislocation [4]. Liu et al. also found the enlarged posterior medial condyle and the shortened posterior lateral condyle in patients with patellar dislocation and trochlear dysplasia [5]. Therefore, deformity of posterior femoral condyle may be widely distributed in patients with patellofemoral arthritis.

Recent studies by Zhao et al. found that the main contributors to increased femoral anteversion in patients with patellar dislocation were the middle and distal femur [6], and other authors also discovered that the leading contributors to increased femoral anteversion were the middle and distal femur [7, 8]. Chang and Li et al. found that patients with severe valgus knees had greater femoral anteversion and dysplasia of the posterolateral condyle was also a key cause of femoral anteversion [9, 10]. These findings all indicate that the deformity within the posterior femoral condyle plays a significant role in the distal femoral torsion.

Meantime, the shape of the posterior femoral condyle is closely related to the tibiofemoral movement pattern and the movement track of the patellofemoral joint during the knee flexion, which is crucial for the change of the pressure of the patellofemoral joint [11]. Dai et al. have recently reported that patellar tilt, patellar shape, and petella alta are closely related to the severity of patellofemoral arthritis [12]. Shuhei Otsuki and others found that the severity of patellofemoral arthritis in varus knee was associated with tibial tuberosity-trochlear groove distance(TT-TG) and patellar tilt [13]. Furthermore, P. Abadie et al. found that the posterior femoral condyle angle would affect the degree of patellar tilt [14]. Patellar tilt is an important factor for patellofemoral arthritis. It remains unknown whether the morphological changes of the posterior femoral condyle affect the severity of patellofemoral arthritis.

Therefore, there are two aims in this study. One is to investigate the correlation between the distal femoral torsion and the severity of patellofemoral arthritis by assessing the morphological changes of the posterior femoral condyle. And the other is to evaluate the correlation between the distal femoral torsion and TT-TG and patella tilt. Our study hypothesizes that the more greatly the distal femur rotates inward, the severer PFOA would become.

## Methods and materials

This study has been approved by the Ethics Committee of our hospital (Number: Z2022-004–3; Date:2022–04-10). A retrospective analysis was conducted on imaging data of 125 patients with PFOA that had been admitted to our hospital from January 2020 to March 2021. All cases chosen in the study were hospitalized patients with osteoarthritis who had undergone either the total knee arthroplasty or patellofemoral joint arthroplasty. Table 1 shows the demographic data of 125 participants.

**Table 1:** Patients demographics

Characteristic	PFOA group
Knees	125
Mean age, yrs (SD; range)	65.78(6.61;52–79)
Female:male, n,(%)	79(63):46(37)
Mean BMI, kg/m^2^ (SD; range)	25.35(2.70;21–30)
Side, left/right,n(%) PFOA grade,n(%)	59(47):66(53)
I	39(31)
II	40(32)
III	46(37)

Inclusion criteria:

1. The PFOA patient group included in our study must be diagnosed with PFOA before surgical operation,
2. patients should receive CT and lower-extremities digital radiography and knee joint X-ray examinations.

Exclusion criteria for this study included:

1. traumatic PFOA,
2. patients without CT or lower-limb digital radiography, knee-joint X-rays imaging,
3. patients with DDH (developmental dysplasia of the hip, central edge angle < 20°),
4. medical history of lower-extremity surgery.

### PFOA imaging assessment

PFOA was evaluated by two orthopedic surgeons, and CT Imaging was used. According to Merchant et al. [15], the orthopedic surgeon evaluated PFJ joint space narrowing at stages 1–4. Stage 1 was mild (the joint space was 3 mm at least). Stage 2 was moderate ( the joint space is less than 3 mm and free of bone contact). Stage 3 is severe, and some bone contact is less than 1/4 articular surface. Stage 4 is very severe, and the articular surfaces of the articular bones are completely in contact with each other. We classified both Stage 3 and Stage 4 as “Stage-3 severe patellofemoral arthritis”, which meant that severe PFOA was identified as long as obvious bone-to-bone imaging was discovered. The evaluation of PFOA was mainly based on the lateral PFOA, as the occurrence of lateral patellofemoral arthritis was more consistent with the shape of femoral condyle of patellar dislocation reported in previous studies.

### Evaluation of distal femoral torsion and femoral condyle morphology

**Fig. 1:**
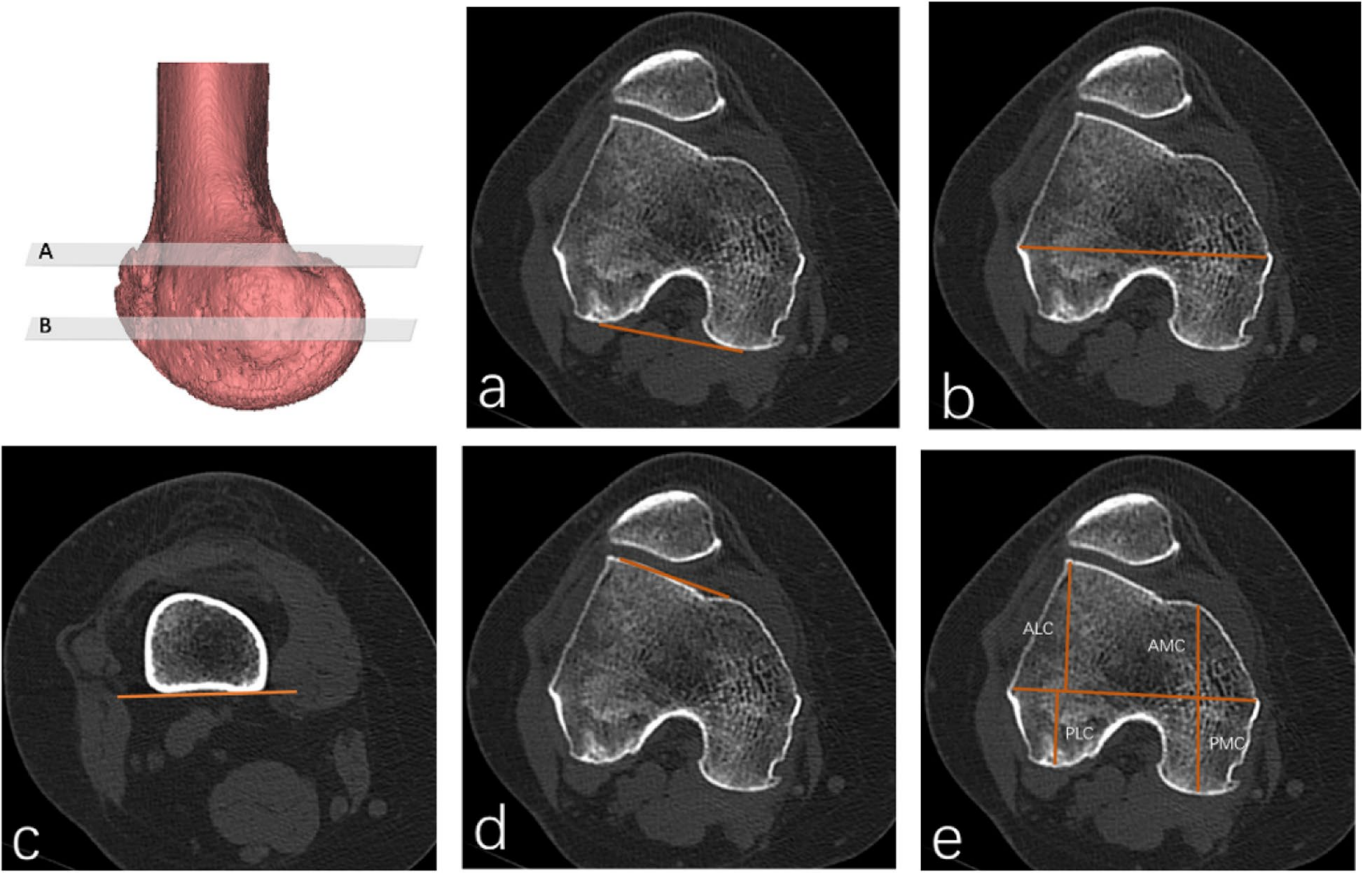
Distal femoral torsion was measured on transverse CT scan at different levels. Two lines are drawn to measure the (**A**) distal femur torsion (distal femur–posterior condyles), (**B**) and posterior femoral condyle torsion (clinical transepicondylar axis–posterior condyles). **a** The PCL is defined as the line connecting the the posterior margin of the lateral and medial femoral condyles. **b** The CEA is defined as the line passing through the apexes of the medial and lateral femoral epicondyles. **c** The DFL is defined as the tangent of the posterior part of the distal femur at the upper level of the popliteal fossa. **d** The ACL is defined as the tangent line connecting the anterolateral condyle of the femur to the apex of the anteromedial condyle. **e** Diagram of ALC,AMC,PLC and PMC

Distal femoral torsion was radiologically measured. Figure 1 shows the transepicondylar axis (TEA) and posterior condyle line (PCL). The distal femur line (DFL) is defined as the tangent of the posterior part of the distal femur, located at the upper level of the popliteal fossa. The TEA is defined as the line passing through the apexes of the medial and lateral femoral epicondyles. The PCL is defined as the line connecting the posterior margin of the lateral and medial femoral condyles. The anterior condyle line (ACL) is defined as the line passing through the anterior lateral condyle of the femur and the apex of the anterior medial condyle. The distal femoral torsion angle (DFL-PCL) is defined as the angle between DFL and PCL. The torsion angle of posterior femoral condyle (TEA-PCL) is defined as the angle between TEA and PCL. Meanwhile, the angle between the distal femur and the transepicondylar axis (DFL-TEA) is defined as the angle between DFL and TEA. The torsion angle of the anterior femoral condyle (TEA-ACL) is defined as the angle between TEA and ACL.

Axial images of the distal femur on the section of the "Roma Dome" (circular inter-condyle notch) were selected to show the complete shape of the femoral trochlea and femoral condyle. The length of the anterior and posterior condyles is defined as the vertical distance between the apex of the condyle cortex and the TEA. If anteromedial condyle dysplasia is severe, measurements are taken at the medial inflection point of the trochlea or at the top of the inclined plane. To eliminate the individual differences, the ratio of the anterior medial condyle (AMC%), posterior medial condyle (PMC%), anterior lateral condyle (ALC%), and posterior lateral condyle (PLC%) in the medial and lateral condyles were evaluated (Fig. 1).

### Evaluation of TT-TG, Patellar tilt and petellar height

**Fig. 2:**
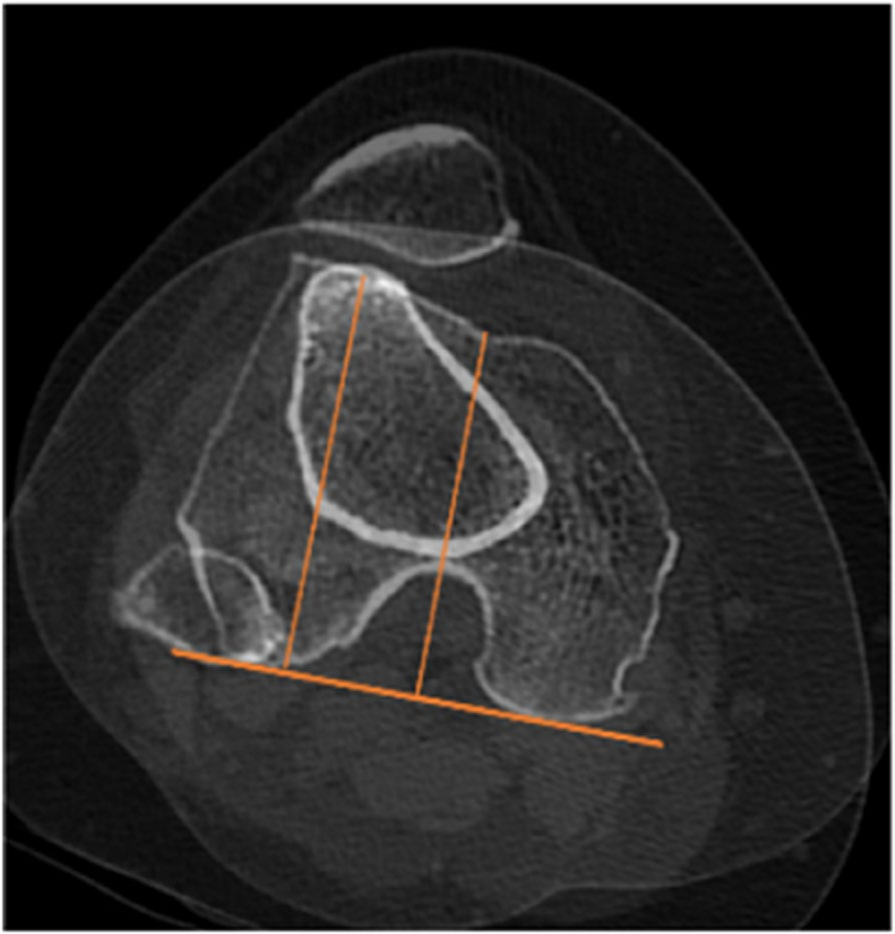
The TT-TG distance is defined as the distance measured on CT between the most anterior part of the tibial trochanter and the middle of the trochlear groove

**Fig. 3:**
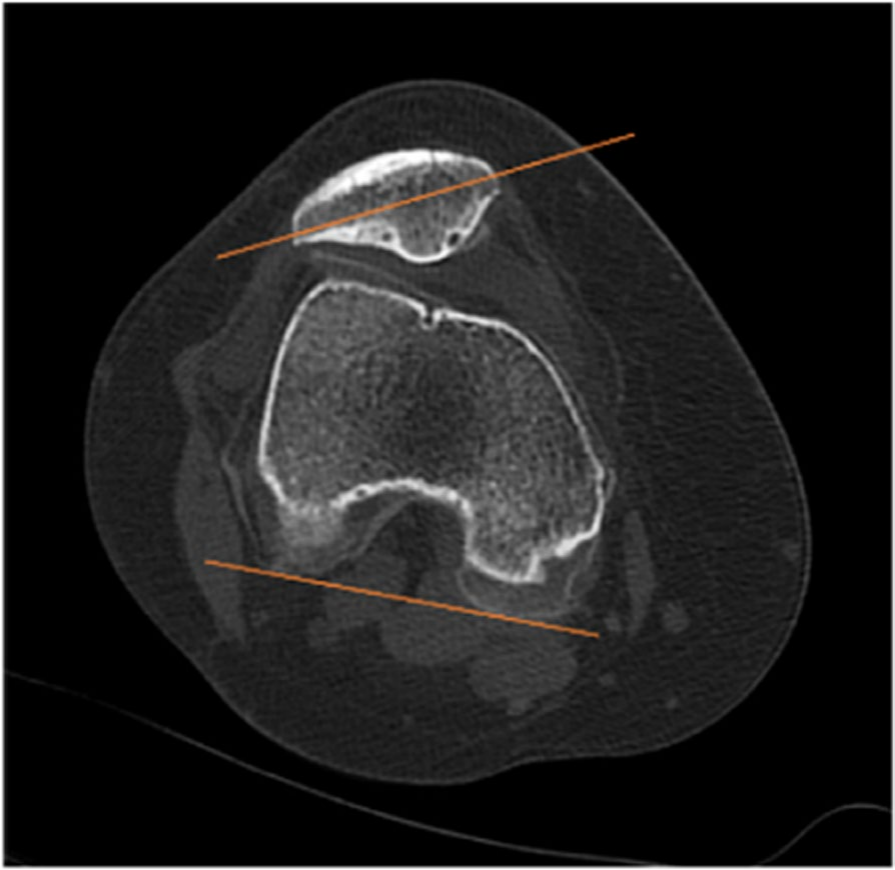
The patella tilt angle is the angle between the line passing through the long axis of patella and PCL

TT-TG distance is defined as the distance measured on CT between the most anterior part of the tibial trochanter and the middle of the trochlear groove (Fig. 2). The patella tilt angle is the angle between the line passing through the long axis of patella and PCL (Fig. 3). The height of patella was evaluated by canton index on lateral radiographs: distance from distal articular surface of patella to anterior edge of tibial plateau/length of articular surface of patella [16].

### Statistical analysis

All statistic data were calculated by using SPSS v.24.0 (IBM, Armonk, New York, USA). The effectiveness of the method was verified by recording the intra-group and inter-group correlation coefficients of 50 cases. All measurements were taken separately by two orthopaedic surgeons who were unaware of the results. The Pearson correlation coefficient was used to analyze the correlation between TT-TG, patellar tilt, femoral condyle ratio and DFL-PCL, DFL-TEA, TEA-PCL. The value is within the range of -1 ~ 1, very good (0.6 ~ 0.8), good (0.4 ~ 0.6) and poor (0.2 ~ 0.4) respectively. Data were shown as mean ± standard deviation (SD). The multiple logistic regression (Level I as the reference level) was used to explore the correlation between DFL-PCL, DFL-AEA, TEA-PCL and the radiographic severity of lateral PFOA. To avoid the effect of patellar height, TT-TG and patellar tilt on the results, CT and X-rays were used to evaluate these indicators respectively and the logistic regression model was applied to obtain the OR value. A P value lower than 0.05 was considered statistically significant.

## Results

The inter-group correlation coefficients of the two observers ranged from 0.816 to 0.997, indicating good consistency. With the increased severity of PFOA, TT-TG, patellar tilt, DFL-PCL, DFL-TEA, and TEA-PCL all tended to increase (Table 2). It was found that patellar tilt was correlated with DFL-TEA and TEA-PCL and that TT-TG was not significantly correlated with distal femoral torsion. At the same time, the distal femoral torsion was correlated with the proportion of the lateral femoral condyle, suggesting that the increase of the anterolateral femoral condyle and the shortening of the posterolateral femoral condyle led to the change of the axis of the femoral condyle, which increased the internal rotation of the distal femur and affected the alignment of the patellofemoral joint (Table 3).

**Table 2:** Results of radiological parameters of femur

Parameters	PFOA1	PFOA2	PFOA3	F	P
	(*n* = 39)	(*n* = 40)	(*n* = 46)		
TT-TG	11.97 ± 5.27	14.67 ± 4.35	16.06 ± 5.07	7.407	**0.001**
Patella tilt	11.64 ± 5.33	12.66 ± 5.69	17.82 ± 6.07	14.52	** < 0.001**
Patella height	1.21 ± 0.09	1.16 ± 0.10	1.18 ± 0.08	2.178	0.118
DFL-PCL	7.52 ± 3.13	8.95 ± 3.42	10.86 ± 2.65	12.77	** < 0.001**
DFL-TEA	2.25 ± 4.11	2.77 ± 2.97	4.29 ± 2.97	4.268	**0.016**
TEA-PCL	5.24 ± 1.81	6.21 ± 2.17	6.77 ± 1.99	6.233	**0.003**
TEA-ACL	11.00 ± 3.32	11.26 ± 2.87	10.46 ± 3.29	0.705	0.496

**Table 3:** Pearson correlation analysis of femoral condyle morphology and distal femoral torsion parameters

	DFL-PCL	DFL-TEA	TEA-PCL
ALC%	-0.073	-0.201^*^	0.231^*^
PLC%	0.073	0.201^*^	-0.231^*^
AMC%	0.002	0.086	-0.116
PMC%	-0.002	-0.086	0.116
TT-TG	0.074	0.056	0.046
Patella tilt	0.243^*^	0.113	0.205^*^

The“*” means statistical significance. *P* < 0.05

At the same time, the four indexes related to distal femoral torsion were analyzed by logistic regression. The results showed that compared with PFOA I patients, DFL-PCL, DFL-TEA and TEA-PCL were risk factors for the increased severity of patellofemoral arthritis in PFOA III patients. There was no significant difference in PFOA II patients. (Table 4).

**Table 4:** Relationship between distal femoral torsion and lateral PFOA radiographic severity (PFOAI grading as the reference category)

PFOA Grade	Parameters	P	OR	95% confidence interval	
II	DFL-PCL	0.069	1.164	0.988	1.372
	DFL-TEA	0.674	1.030	0.896	1.185
	TEA-PCL	0.052	1.271	0.998	1.619
	TEA-ACL	0.605	1.039	0.899	1. 200
III	**DFL-PCL**	** < 0.001**	**1.493**	**1.214**	**1.835**
	**DFL-TEA**	**0.014**	**1.226**	**1.041**	**1.443**
	**TEA-PCL**	**0.008**	**1.433**	**1.100**	**1.866**
	TEA-ACL	0.929	0.993	0.848	1.163

## Discussion

**Fig. 4:**
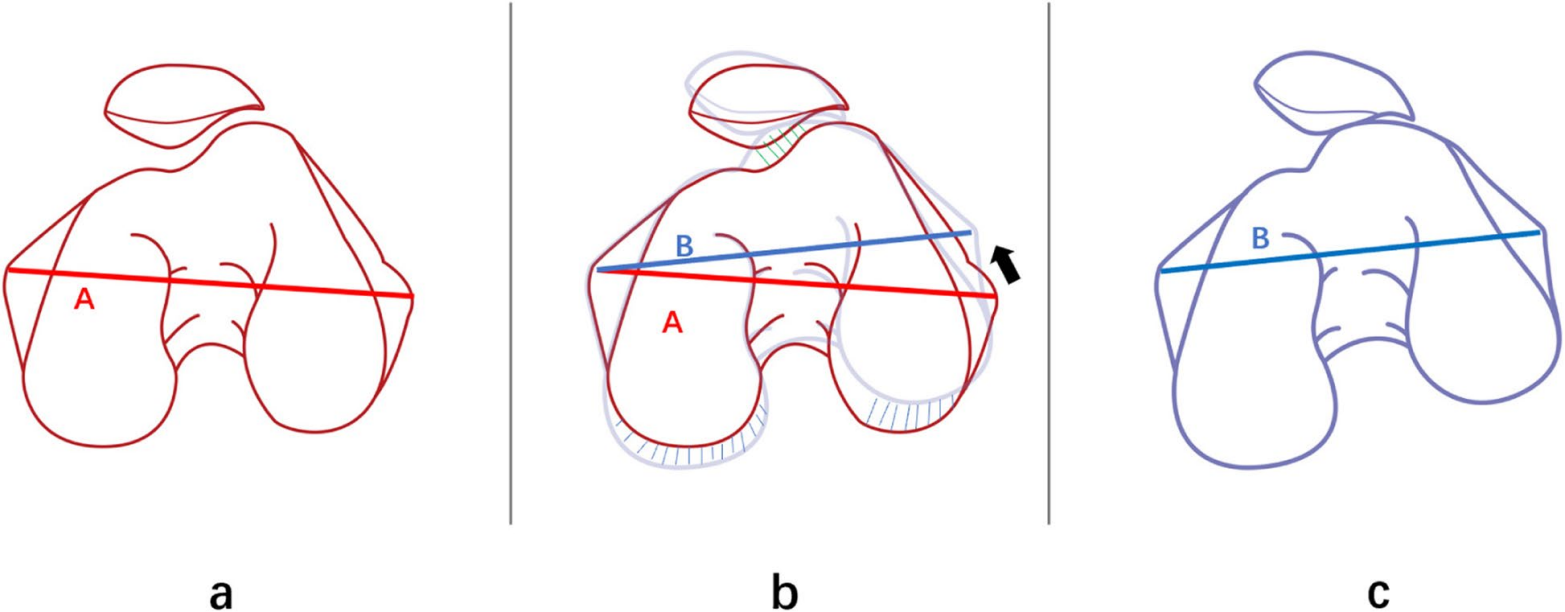
**a** to **c** represents the course of abnormal changes in the femoral condyle. Line A to line B indicates the change in axial torsion of the femoral condyle. **b** The blue dashed line indicates the enlargement of the posterior medial condyle of femur and the shortening of the posterior lateral condyle, showing the proportion change of the posterior condyle of both the inner and outer sides of femur. The green dashed line indicates an inward turning projection of the anterolateral condyle

The most important finding of our study was that the severity of PFOA was associated with the distal femoral torsion. The increase of TEA-PCL reflects the relative change of the PMC and PLC and the increase of torsion of the posterior condyle of the femur. The increase of the ALC and the shortening of the PLC lead to the increase of torsion of the transcondyle axis of the femoral condyle, which together contribute to the increase of distal femoral torsion (Fig. 4). This suggests that there is an abnormal transcondyle axis and posterior condyle of the femur in patients with patellofemoral arthritis. The influence of this abnormal change on the patellar trajectory should be recognized during total knee arthroplasty, as it provides additional considerations for the selection of rotational femoral osteotomy in total knee arthroplasty.

Distal femoral deformity is a main contributor to the femoral torsion. Omer Faruk Erkocak et al. found that rotational deformity of the lower extremity increases the risk of anterior knee pain [17]. Liu et al. found that the morphological deformity of the distal femur may be an important factor for the lower-limb rotation [5]. Dagneaux and Liska et al. demonstrated that internal torsion of the distal femur resulting from the increased femoral anteversion would increase pressure on the patellofemoral lateral articular surface [18, 19]. Our study also confirmed that the internal rotation of the distal femur is related to the severity of PFOA.

Julien Roger et al. believed that the increased internal rotation of the distal femur was due to the ratio imbalance between the PLC and PMC, which ultimately led to the increase of PCA and this change might also affect the position of the patella [4]. We also found that the patella tilt was related to the torsion of the posterior femoral condyle. In addition, we found that changes of the femoral condyle were not only limited to the posterior femoral condyle. When we measured DFL-TEA with DFL as the baseline, we found that the axis of the femoral transcondyle had also changed. This is caused by the ratio imbalance of the lateral condyle, the protrusion of the anterolateral condyle and the shortening of the posterolateral condyle, eventually resulting in the internal rotation of the femoral transcondyle axis. Yang et al. have confirmed this finding in patients with trochlear dysplasia [3], but we are the first to demonstrate that in patients with patellofemoral arthritis. TEA has been widely accepted as a stable reference index for femoral axial rotation [20]. However, our study found that patients with patellofemoral arthritis may be accompanied by dysplasia of the PLC and have TEA different from those with tibiofemoral arthritis. In this case, when performing TKA surgery, posterior condylar osteotomy with the femoral transcondylar axis as the reference may not improve the abnormal patellar trajectory. Thienpoint et al. mentioned that during femoral osteotomy, patients with patellofemoral arthritis require external rotation more than average to restore normal patellar trajectory [21]. Lim et al. reported that when there is significant PLC degeneration, there will still be internal rotation of the femoral prosthesis after placement, which may lead to symptoms of patellofemoral joint in the future [22]. All of these suggest that we need to perform more external rotational femoral osteotomies to improve patellar trajectory, not just rotational osteotomy parallel to the transcondylar line. Certainly, excessive external rotation osteotomy may lead to unbalanced medial gap and lateral gap, especially the relaxation of the medial gap, which is unacceptable in surgery. This forces us to perform the lateral retinacular release during the operation to adjust the patella trajectory and reduce the potential for future patellofemoral complications. In addition, internal rotation of the distal femur may also be caused by the change of femoral anteversion [10]. For patients with significantly increased femoral anteversion, whether we can adopt distal femoral derotation osteotomy combined with TKA still needs further exploration.

The shape of femoral condyle plays a key role in knee flexion. When the knee flexes, different sizes of medial and lateral femoral condyles lead to external rotation of the femur on the tibia [11]. Yuichi et al. reported that increased CTA would lead to the increase in the internal rotation of the tibia and relative external rotation of the femur [23]. Therefore, in theory, patients with PLC hypoplasia may experience excessive rotation during flexion, resulting in patellar tilt or subluxation [4, 24]. Bigert et al. also reported the instability of knee flexion due to PLC degeneration, resulting in patellar instability [25]. P. Abadie et al. reported that increased PCA would lead to a mismatch between the patella position and the center of the femoral condyle, resulting in patellar tilt or subluxation [14]. Therefore, patellar instability during knee flexion caused by the shortening of the posterolateral femoral condyle increases the risk of patellofemoral arthritis. Protruding ALC and excessive internal rotation of the distal femur are important factors for increased contact pressure of the lateral condyle and aggravated lateralization of the patella [26].

**Fig. 5:**
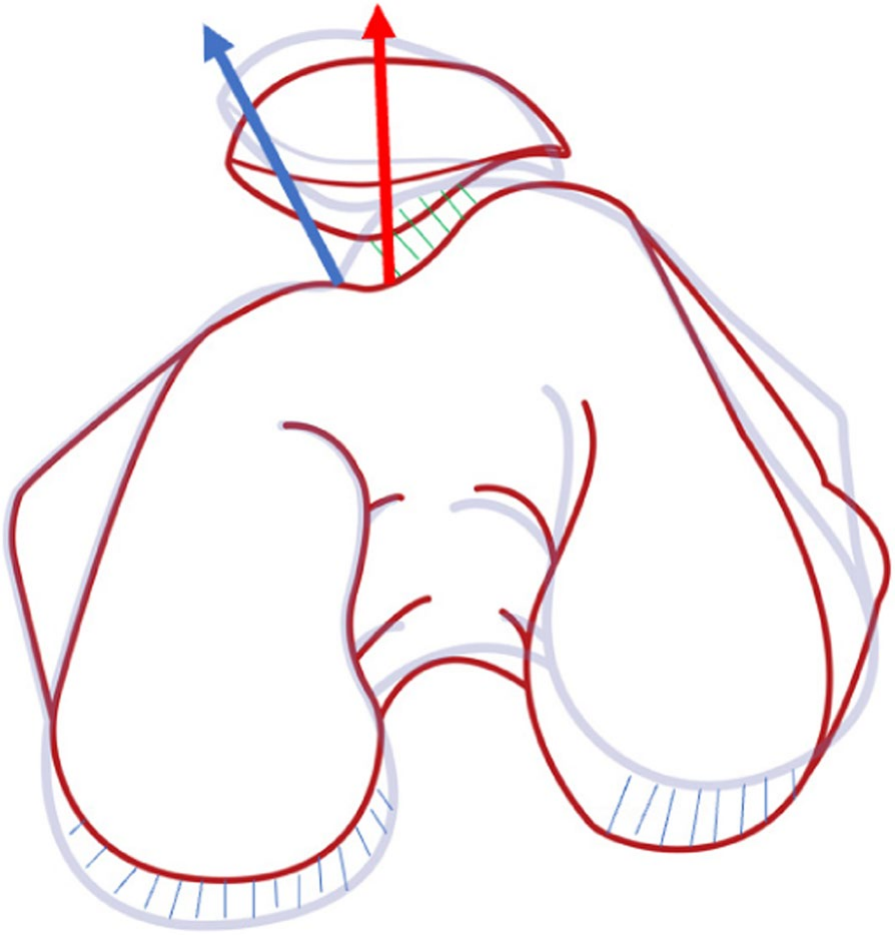
The orientation of the femoral trochlear does not match the patella position

Julien Roger et al.once questioned whether the absolute length of the shortened PLC could have a clinical effect, and he believed that the relative change between PMC and PLC was the cause of the poor patellar trajectory [11]. Dominic Gillespie et al. also reported that the shortening of the isolated posterior femoral condyle appeared not to be associated with patellar instability [24]. Our study revealed that both DFL-PCL and TEA-PCL correlated with patellar tilt. We believed that variation of the shape of the femoral condyles would result in the increase of the internal torsion of the femoral transepicondylar axis and posterior condylar torsion. Due to that, the patella mismatched with the orientation of the femoral trochlea and the patellar tilt increased, contributing to the deterioration of patellofemoral arthritis (Fig. 5).

In addition, our study found no correlation between TT-TG and the shape of distal femur. TT-TG is affected by many factors, such as femoral torsion, tibial torsion and external placement of tibial nodules. The simple distal femoral torsion can not considerably affect TT-TG. Shuntaro Nejima et al. and Shuhei Otsuki et al. reported that PFOA was associated with TT-TG and patellar tilt [13, 27]. This suggests that in treating patients with patellofemoral arthritis and excessive enlargement of TT-TG, surgeons should not rely solely on the rotation of femoral prosthesis to reduce TT-TG, as this attempt may not be very effective, and other surgical methods should also be used to correct the patellar trajectory.

There are some limitations to our study. First, it is a pity that we did not include FAA into this study. Due to the failure to obtain whole-lower-limb CT data of patients with knee osteoarthritis, our study lacked a controlled evaluation of proximal femoral torsion. Even so, taking the deformity within the distal femur into consideration is very important for the treatment of patellofemoral arthritis, as it plays an important role in selecting TKA for osteotomy, gap balance and adjustment of patellar trajectory for patients with end-stage osteoarthritis. Second, in our study, the patellofemoral joint was mainly considered and patients without DDH were targeted. Our conclusions may not be applicable to patients with DDH, as there may be proximal femoral deformity within them. In the future, we will continue to study the influence of high torsion of both proximal femur and distal femur on patellofemoral joint. Third, there exist a variety of risk factors for the bone structure of the patellofemoral joint. We assessed only some risk factors of interest and other pathological factors may interfere with our final results, but our study provides a new reference for understanding the correlation between the distal femoral torsion and patellofemoral arthritis.

## Conclusion

The severity of PFOA correlates with the distal femoral torsion. A change in the proportion of the femoral condyle results in the variation in the axis of the femoral transcondyle, especially the lateral condyle. In clinical practice, more attention should be paid to the impact of the change of femoral condyle axis on the patellofemoral joint.

## Supplementary Information

**Additional file 1.**

## Data Availability

The detailed data and materials of this study are available from the corresponding author via e-mail on reasonable request.
