# Supplementary material for: Study on the correlation between the severity of patellofemoral arthritis and the morphology of the distal femur
Source: BMC Musculoskelet Disord. 2023 Feb 2;24:90. doi: 10.1186/s12891-023-06198-z (PMC9893554; doi:10.1186/s12891-023-06198-z)
Supplement: Supplementary file 1 — Additional file 1. [file 12891_2023_6198_MOESM1_ESM.docx]

Supplementary table. Measurement Methods of The Parameters.

| Parameter | Abbreviation | Definition |
| --- | --- | --- |
| transepicondylar axis | TEA | The TEA is defined as the line passing through the apexes of the medial and lateral femoral epicondyles. |
| posterior condyle line | PCL | The PCL is defined as the line connecting the the posterior margin of the lateral and medial femoral condyles. |
| distal femur line | DFL | The distal femur line (DFL) is defined as the tangent of the posterior part of the distal femur, located at the upper level of the popliteal fossa. |
| anterior condyle line | ACL | The anterior condyle line (ACL) is defined as the line passing through the anterior lateral condyle of the femur and the apex of the anterior medial condyle. |
| distal femoral torsion angle | DFL-PCL | The distal femoral torsion angle is defined as the angle between DFL and PCL. |
| torsion angle of posterior femoral condyle | TEA-PCL | The torsion angle of posterior femoral condyle (TEA-PCL) is defined as the angle between TEA and PCL |
| - | DFL-TEA | The angle between the distal femur and the transepicondylar axis is defined as the angle between DFL and TEA. |
| torsion angle of anterior femoral condyle | TEA-ACL | The torsion angle of anterior femoral condyle is defined as the angle between TEA and ACL. |
| - | AMC% | the ratio of the anterior medial condyle in the medial condyle |
| - | PMC% | the ratio of the posterior medial condyle in the medial condyle |
| - | ALC% | the ratio of the anterior lateral condyle in the lateral condyle |
| - | PLC% | the ratio of the posterior lateral condyle in the lateral condyle |
